# Commentary: Systems Biology and Its Relevance to Alcohol Research

**Published:** 2008

**Authors:** Q. Max Guo, Sam Zakhari

**Keywords:** Alcohol-induced disorders, alcohol research, biomedical research, systems biology, biological systems, mathematical modeling, genomics, epigenomics, transcriptomics, metabolomics, proteomics

## Abstract

Systems biology, a new scientific discipline, aims to study the behavior of a biological organization or process in order to understand the function of a dynamic system. This commentary will put into perspective topics discussed in this issue of *Alcohol Research & Health*, provide insight into why alcohol-induced disorders exemplify the kinds of conditions for which a systems biological approach would be fruitful, and discuss the opportunities and challenges facing alcohol researchers.

Until recently, most biologists’ efforts have been devoted to reducing complex biological systems to the properties of individual molecules. However, with the completed sequencing of the genomes of humans, mice, rats, and many other organisms, technological advances in the fields of high-throughput genomics^1^ and functional genomics^2^ have generated enormous amounts of information on the properties of genes, RNAs,^3^ proteins, and metabolic products (i.e., metabolites) in an organism. The billions of data points generated by these high-throughput studies are far beyond the reach of reductionist approaches. High-throughput technologies have offered biologists tremendous opportunities but also have created considerable challenges. How can we take advantage of this wealth of information to understand its biological significance in health and disease? Systems biology is an emerging discipline that deals with, and takes advantage of, these enormous amounts of data. Although scientists and engineers have applied the concept of an integrated systemic approach for years, systems biology has only emerged as a new, distinct discipline to study complex biological systems in the past several years. A database search using the phrase “systems biology” in the ISI Web of Science has found only 3 publications in 2001; in 2006, this number had reached 575 (see figure 1).

With the emergence of this powerful new discipline, we are tempted to ask the following questions: Is systems biology suitable for alcohol research? What kinds of alcohol-related problems can we address using a systems biology approach? What opportunities and challenges are there in current and future research? These are the kinds of questions that the articles in this special systems biology issue of *Alcohol Research & Health* intend to address. In this commentary, we will try to put the topics discussed in this issue into perspective, provide views on the significance of systems biology for alcohol research, and discuss opportunities and challenges facing alcohol researchers.

## What Is Systems Biology?

Systems biology is a new scientific discipline that studies the behaviors of complex biological organizations or processes through the integration of diverse quantitative information and mathematical modeling to generate a predictive hypothesis on the functions of the dynamic biological system (Aderem 2005; Auffray et al. 2003; Hood et al. 2004; Kirschner 2005; Liu 2005; O’Malley and Dupre 2005; Weston and Hood 2004).

Systems biology may have quite different meanings to different people. In general, systems biologists can be organized into two camps. In the first camp, the “systems-theoretic biologists” think that the focus of systems biology is to elucidate system principles and properties of operation based on component interactions in the biological system (Bork and Serrano 2005; O’Malley and Dupre 2005). To them, systems biology is very abstract and precise. The mere integration of constituents in the system for understanding the emergent properties^4^ of component interactions is insufficient for these theory-oriented systems biologists. However, the majority of today’s systems biologists, who can be described as “pragmatic systems biologists,” are gathered in the other camp (O’Malley and Dupre 2005). They use systems biology as a practical but vague term, denoting the study of interconnected phenomena as systems and the collective analysis of large amounts of diverse data by integration and modeling. Despite these differences, the pragmatic and theoretic systems biologists share some common ground. For example, both agree that systems biology involves data integration and mathematical modeling and that models generated through systems biology studies should be predictive.

Alcohol-induced disorders are very complex, involving numerous pathways and tissues simultaneously. Because the approaches taken by theory-oriented systems biologists are very abstract and require specific expertise in computation and modeling, early systems biologists in the field of alcohol research will most likely adopt the pragmatic approach.

## What Is a Biological System?

“Biological system” is a loosely defined term. To understand what constitutes a biological system, it is helpful to revisit a visionary paper by Hartwell and colleagues (1999) published before systems biology became a household term for biologists. Although the authors did not mention the phrase “systems biology” in that paper, their proposed concept of functional modules provides the building blocks for defining a biological system. Hartwell and colleagues proposed that the functions of a cell are carried out by modules made of many different kinds of interacting molecules. They argued that these functional modules provide a critical level of biological organization. Functional modules are not a mere collection of components. Rather, they are derived and shaped by millions of years of evolutionary selection for survival, fitness, and reproduction. For example, the mitotic spindle^5^ in a cell is not just created from simple polymerization of α- and β-tubulins.^6^ It involves the interaction of the polymerized microtubules with motor proteins and the communication with many other cellular signals to accurately segregate the sister chromosomes. The emergent properties during chromosome segregation cannot be achieved by the collective properties of tubulins, microtubules, motors, or chromosomes alone. The spindle can be considered a functional module, which communicates and interacts with and organizes many other molecules and structures. The amazing harmony of spindle behaviors during cell division is achieved through evolutionary selection, as evidenced by comparing the spindle formations during closed mitosis and open mitosis. During closed mitosis in yeast, the spindle is formed within the nuclear membrane, which does not break down during mitosis. However, in higher organisms, the nuclear membrane breaks down during mitosis and the spindle is formed without constrains of the nuclear membrane (open mitosis). The function of the nuclear membrane for spindle formation in yeast may have evolved into that of a new structure called the spindle matrix in higher organisms (Tsai et al. 2006; Zheng and Tsai 2006).

A functional module always possesses emergent properties derived from the interactions of individual components. These discrete properties could not be predicted by studying the individual components in isolation. Although a functional module is a distinct entity separable from the functions of other modules, it is not necessarily a rigid and fixed structure. A component in one module also could belong to another module. In addition to being insulated from each other in order to carry out many diverse functions, functional modules are interconnected. The interactions of these interconnected modules in turn generate a higher layer of functions at another biological level.

Recognizing functional modules as the building blocks of a biological organization or process, we could define a biological system with the terms used in modular biology. A biological system consists of either a single functional module or many interconnected modules that work in concert to carry out the functions of a biological organization or process. To illustrate this point, we will use mitochondria as an example. The mitochondrial electron transport chain^7^ consists of five enzyme complexes. Complex I (NADH-coenzyme Q reductase) contains 45 subunits; Complex II (succinate-coenzyme Q reductase) contains 4 subunits; Complex III (coenzyme Q-cytochrome c reductase) has 11 subunits; Complexes IV (cytochrome c oxidase) and V (adenosine triphosphate [ATP] synthase) have 13 and 16 subunits, respectively. Each of these complexes can be considered a functional module, consisting of various interacting subunits. But each of these complexes also can be considered a biological system if our goal is to understand how each of the complexes works. However, if our goal is to understand how ATP is generated through oxidative phosphorylation,^8^ we would consider the entire electron transport chain as a biological system. In this case, the five complexes would be considered interconnected modules in the system. With the same logic, we can consider the mitochondrion, the cell, organs, and organisms as larger biological systems with increasing complexity. The functional modules are interconnected in a hierarchical structure to form subsystems and systems.

If we use the systems biology approach to study how Complex I functions, we would gather information on the identity of each component; the regulation of its gene expression; its synthesis, degradation, and transport; and its interaction with other subunits, membranes, and molecules, etc. In addition, we also would need to collect cellular, physiological, and clinical measurements associated with the perturbation of the complex. We also could collect data on the structural and behavioral alterations of the complex in response to external cues. These data then could be integrated and computed mathematically to generate a model of how Complex I functions. Based on this model, we should be able to hypothesize and predict how the function of the complex would change if we perturb a specific component in the system, say by ethanol. For example, if the amount of one nuclear-encoded subunit is rate limiting for assembly of the complex, one may predict that the expression of this nuclear gene is tightly regulated. Aberrant regulation of its gene expression (e.g., by a mutation of a transcriptional inhibitor) could cause an increased amount of the subunit and thus increased activity of NADH dehydrogenase (Complex I) in the mitochondria. When experimentally tested, this prediction could prove incorrect, because the overexpressed subunit also could be regulated through degradation or transport into mitochondria. Based on the new experimental data, the model would be further refined and improved. This integration of experimentation, data processing, and mathematical modeling is an iterative process, which continues until a relatively detailed and sufficiently accurate model is achieved.

Despite different levels of complexity in various biological systems, they all share some common characteristics (Aderem 2005; Westerhoff and Palsson 2004). First, all biological systems display some forms of modularity. These modules are interconnected, often in a hierarchical structure. Second, a biological system always possesses some emergent properties. The study of any participating component alone would not be able to predict these new properties. For example, studies of a subunit in mitochondrial Complex I can never predict how the complex is assembled and how NADH dehydrogenase functions. Similarly, studies of Complex I alone can never predict how ATP is generated through the electron transport chain. Finally, biological systems are robust. A biological system is not merely a collection of its parts. As described in the above example, the mitotic spindle, as a biological system, is not formed through simple polymerization of tubulins. A dysfunctional spindle would cause detrimental consequences for the cell, such as cancer or apoptosis. A biological system is a survivor of furious competition and natural selection over millions of years. Over time, organisms have evolved to develop many complex mechanisms, such as positive and negative feedback, to maintain the functional stability of their biological systems. Therefore, it is no surprise that our biological studies often amaze us with the robustness and harmony of biological systems.

## Alcohol-Induced Disorders Are Systems Biology Diseases

Alcohol’s actions in our body are very complex, and alcohol-induced physiological or behavioral changes are affected by a variety of intrinsic and environmental factors. Alcohol-induced disorders, including organ damage and addiction, reflect the genetic and epigenetic^9^ makeup of an individual and the cumulative responses to alcohol exposure and environmental perturbations over time. Each of these factors may contribute only a small fraction to the symptoms or phenotypes. At the molecular level, the effects of alcohol and its metabolites are the consequences of changes in DNA, RNA, proteins, metabolites, and other molecules. At the systems level, alcohol affects a variety of organs, biochemical or signaling pathways, and other biological processes. This high degree of complexity in alcohol-induced disorders renders the traditional gene-by-gene or single-discipline studies limited because they only provide a fragmented view of a very complex picture. An integrated approach, such as systems biology, is essential in revealing a global picture of the perturbations leading to disease. From either pathogenetic or etiological perspectives, alcoholism is indeed a systems biology disorder. Alcohol research using a systems biology approach will prove fruitful in unraveling the mechanisms of complex alcohol-induced disorders.

## Components of Systems Biology for Alcohol Research

**Figure 2:**
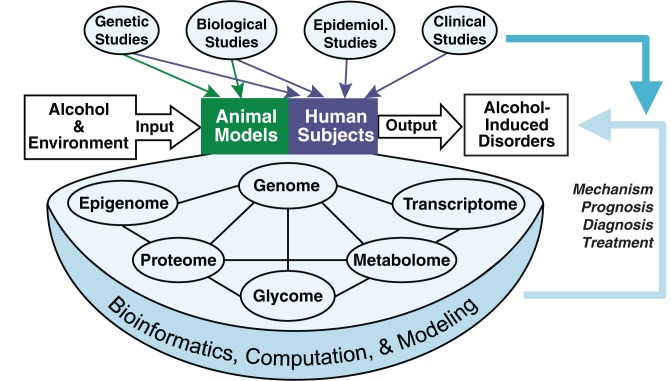
Systems biology approach for alcohol research. The diagram emphasizes the concept of integration and the significance of bioinformatics, computation, and mathematical modeling. The lines between the “omes” are meant to indicate that the web of data on all the “omes” should be integrated and collectively analyzed. Genomic and functional genomic data also should be integrated with genetic, other biological, epidemiological, and clinical studies. The ultimate goal of systems biology studies is to understand the mechanisms and develop better prognosis, diagnosis, and treatment for alcohol-induced disorders.

Genomics and functional genomics are the driving forces behind the systems biology approach for alcohol-induced disorders, as discussed in other articles in this issue (see figure 2). At the DNA level (genome and epigenome), various global genomic technologies and information can be applied to study genetic variation, gene mutation, gene mapping, and genetic or epigenetic regulation. Both genetic and epigenetic mechanisms are crucially responsible for susceptibility, initiation, progression, and pathogenesis of alcohol-induced disorders. Evidently, approaches to the study of gene regulation purely based on gene sequence would not be sufficient to explain alcohol-induced pathogenesis. A better understanding of epigenetic mechanisms will complement information obtained from genetic, genomic, and functional genomic studies. The information can be integrated with other experimental and clinical measurements to identify complex systems-level responses to alcohol and environmental perturbations.

At the RNA level (i.e., transcriptome), DNA microarray and other global genomic technologies can be used to study the quantity of RNAs and their alternative splicing. In the past several years, a significant amount of microarray data has been generated and is reaching the critical mass that is required for systems biology studies.

At the protein level (i.e., proteome), proteomic technologies can systematically study the identity, quantity, modification, localization, interaction, and function of all proteins in a cell, often in a high-throughput manner. The proteomic approaches would extend the power of the global gene expression analyses for the following four primary reasons: (1) RNA expression sometimes does not correlate with protein expression, which is a more critical indicator of the gene activity; (2) the activity of a protein could be significantly modulated by post-translational modification, which cannot be reflected at the RNA level; (3) the function of a gene is executed by its protein product in a specific subcellular compartment; and (4) a protein molecule provides many more targets for regulation (quantity, functional activity, structure, posttranslational modification, localization, etc.), whereas RNAs mostly are targeted for their quantity. In addition, proteins normally are more stable than RNA in both cells and body fluids. The stability and multiple layers of regulation of proteins make them much better candidates as biomarkers or targets for therapeutic interventions.

At the metabolite level (i.e., metabolome), metabolomics involves a detailed quantitative analysis of low–molecular weight metabolites over changing environmental conditions (e.g., alcohol administration) in a biological system. Metabolites are the intermediate and end products of cellular functions, and their levels and modulation are definitive reflections of an organism’s response to genetic or environmental perturbations. The determination of these metabolites can be achieved by using a variety of tools such as mass spectrometry, nuclear magnetic resonance spectroscopy, capillary electrophoresis, and high-performance liquid chromatography, in conjunction with a wide range of bioinformatic, statistical, and computational tools. A virtual snapshot image can be obtained of the myriad of small molecules within the biological system and how these molecules are modulated in individual time frames. Metabolomic studies in alcohol research are complementary to studies of the genome, transcriptome, or proteome, because they can extract latent biochemical information of diagnostic or prognostic value, reflecting actual biological events, and can serve as a sentinel for diseases. Metabolomics also offers approachable solutions because there are far fewer metabolites than genes or gene products (RNAs and proteins) in many organisms studied. For example, in yeast, there are only approximately 600 low–molecular weight metabolites, compared with more than 6,000 genes.

Another emerging area for systems biology is glycomics, which is a global approach to study complex carbohydrates (or glycans) for their structure and function and their interaction with other carbohydrates, proteins, lipids, and nucleic acids. Carbohydrates and their interaction with other molecules are involved in a wide spectrum of cellular functions. Glycomic studies may reveal glycan changes and provide novel avenues for understanding alcohol’s actions, especially on posttranslational modifications of proteins.

For systems biology studies, bioinformatics, computation, statistical analysis, and mathematical modeling are all pivotal for integrating and making sense of large and complex datasets generated through the high-throughput -omic technologies (see figure 2). Through integration and modeling, these studies would allow us to better exploit the complexity of genomic and functional genomic data and to extract their biological and clinical significance. The integration and modeling of diverse information, including other biological and clinical measurements, would vastly enhance the power of any single-discipline approach, help to decipher the mechanisms of alcohol-induced disorders, and provide new avenues for their prognosis, diagnosis, and treatments.

## Challenges and Opportunities

It is clear from the articles in this issue that well-developed systems biology programs are needed in the alcohol field. This is true in many other fields as well, such as pulmonary diseases, diabetes, and aging. Interestingly, of the 575 systems biology papers published in 2006, many were reviews and meeting abstracts. Currently, comprehensive systems biology studies still are quite uncommon in most biological and biomedical fields. Obviously, systems biology approaches are difficult and present a variety of new challenges. This section focuses on a few of these challenges that are relevant to alcohol research.

First, alcohol research still is facing many technological challenges. As discussed above, genomic and functional genomic studies are the driving forces behind systems biology, which demands the availability of sufficient and reliable data from many different technological platforms (also see Ge et al. 2003). Not only do we need to expand our repertoire of technologies to include epigenomic, metabolomic, glycomic, and other –omic approaches, we must also integrate these technologies in systems biology studies. Second, as with all other fields of biomedical research, the bioinformatics, computation, and statistical or mathematical modeling necessary for systems biology studies present even greater challenges. Finally, additional technological obstacles, such as standardization; creation of uniform, systematic vocabularies and systems of annotation; and digitalized output of other biological and clinical measurements need to be addressed (also see Cassman 2005). Because these challenges are not specific to individual investigators, they should be tackled through collaborative and concerted efforts.

In addition to these technological challenges, we need to address some unprecedented organizational challenges. Traditionally, biomedical research is carried out in individual laboratories with relatively focused areas of inquiry. When collaborations are involved, they are normally simple and limited to a few experiments. Even for a very large endeavor such as the Human Genome Project—in which collaborations were extensive—organizational structure was clear and management was relatively straightforward. For example, each chromosome was sequenced by an individual sequencing center and all sequence information was easily combined into a single database. Systems biology has to deal with much more diverse formats of data, many more different technologies, and much greater complexity in terms of both biological system and organizational structure. Experts involved in collaborative systems biology studies most likely reside in different departments, if not different institutions or companies. Investigators need to overcome many intrinsic and extrinsic barriers to form a multidisciplinary team aimed at understanding the function of a biological system. Research institutions and funding agencies also need to take the necessary steps to encourage and facilitate the sharing and team work that are essential for a systems biology approach. A recent change of policy by the National Institutes of Health, which allows multiple principal investigators on a single grant, represents a move in the right direction.

Finally, systems biology studies often are hampered by the lack of trained investigators with interdisciplinary talents and skills. A fascinating aspect of systems biology is that it integrates large amounts of diverse forms of information for mathematical modeling. Therefore, systems biology necessitates the involvement of computer scientists, statisticians, and mathematicians who also are well-versed in biology, as well as biologists who can understand bioinformatics, statistics, and mathematical modeling. The bottleneck of the systems biology approach is the cross-disciplinary training for a new generation of researchers who can tackle the multidisciplinary complexity with ease. Universities and research institutions need to create multidisciplinary training programs. This issue also needs to be addressed by funding agencies.

Despite these challenges, comprehensive systems biology is a realistic prospect now for the studies of alcohol-induced disorders. Systems biology, with the help of continuing technological innovations and multidisciplinary teamwork, provides the integrative approach necessary for the future prognosis, diagnosis, and treatment of alcohol-induced disorders.

## Figures and Tables

**Figure 1:**
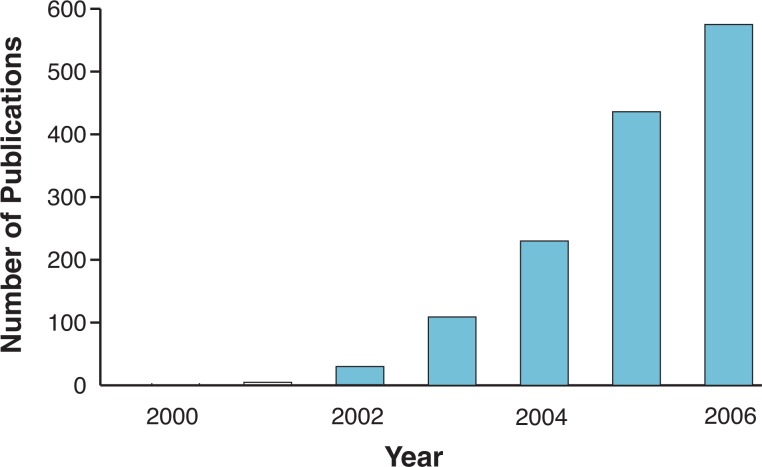
Number of publications with “systems biology” as a key word in the search of the ISI Web of Science. The data plotted are from 2000 to 2006. The Web address for the ISI Web of Science is: http://portal.isiknowledge.com/portal.cgi

## Footnotes

1 High-throughput genomics is the study of the structure and function of an organism’s complete genetic content, or genome, using technology that analyzes a large number of genes at a time. See the glossary, p. 84, for descriptions of other technical terms used in this article.

2 Functional genomics aims to discover, on a large scale, the biological function of particular genes and to uncover how sets of genes and their products work together in health and disease. An example includes gene expression profiling.

3 RNA is mostly involved in the processes of translating genetic information from DNA into proteins. Some non-coding RNAs also are directly involved in other biological processes in the cell.

4 An emergent property is the new characteristic generated through the interaction of individual components.

5 The mitotic spindle is an assembly of microtubules, structural components in the cell, created during the process of cell division known as mitosis.

6 α- and β-tubulins are the proteins that make up microtubules.

7 The mitochondrial electron transport chain is composed of enzymes (Complexes I, II, III, and IV) that transfer electrons and produce a proton gradient, which drives the synthesis of ATP by Complex V.

8 Oxidative phosphorylation is a metabolic pathway that uses energy released by the oxidation of nutrients to produce ATP.

9 Epigenetics is the study of heritable changes in gene function that occur either to DNA without altering its sequences or to DNA-associated histone proteins. These epigenetic changes can either be inherited mitotically from cell to cell or meiotically from generation to generation.

## References

[b1-arh-31-1-5] Aderem A (2005). Systems biology: Its practice and challenges. Cell.

[b2-arh-31-1-5] Auffray C, Imbeaud S, Roux-Rouquie M, Hood L (2003). From functional genomics to systems biology: Concepts and practices. Comptes Rendus Biologies.

[b3-arh-31-1-5] Bork P, Serrano L (2005). Towards cellular systems in 4D. Cell.

[b4-arh-31-1-5] Cassman M (2005). Barriers to progress in systems biology. Nature.

[b5-arh-31-1-5] Ge H, Walhout AJ, Vidal M (2003). Integrating “omic” information: A bridge between genomics and systems biology. Trends in Genetics.

[b6-arh-31-1-5] Hartwell LH, Hopfield JJ, Leibler S, Murray AW (1999). From molecular to modular cell biology. Nature.

[b7-arh-31-1-5] Hood L, Heath JR, Phelps ME, Lin B (2004). Systems biology and new technologies enable predictive and preventative medicine. Science.

[b8-arh-31-1-5] Kirschner MW (2005). The meaning of systems biology. Cell.

[b9-arh-31-1-5] Liu ET (2005). Systems biology, integrative biology, predictive biology. Cell.

[b10-arh-31-1-5] O’Malley MA, Dupre J (2005). Fundamental issues in systems biology. BioEssays.

[b11-arh-31-1-5] Tsai MY, Wang S, Heidinger JM (2006). A mitotic lamin B matrix induced by RanGTP required for spindle assembly. Science.

[b12-arh-31-1-5] Westerhoff HV, Palsson BO (2004). The evolution of molecular biology into systems biology. Nature Biotechnology.

[b13-arh-31-1-5] Weston AD, Hood L (2004). Systems biology, proteomics, and the future of health care: Toward predictive, preventative, and personalized medicine. Journal of Proteome Research.

[b14-arh-31-1-5] Zheng Y, Tsai MY (2006). The mitotic spindle matrix: A fibro-membranous lamin connection. Cell Cycle.

